# Investigating EEG Patterns for Dual-Stimuli Induced Human Fear Emotional State

**DOI:** 10.3390/s19030522

**Published:** 2019-01-26

**Authors:** Naveen Masood, Humera Farooq

**Affiliations:** 1 Electrical Engineering Department, Bahria University, Karachi 75260, Pakistan; 2 Computer Science Department, Bahria University, Karachi 75260, Pakistan; humerafarooq.bukc@bahria.edu.pk

**Keywords:** brain computer interface, classification, common spatial pattern (CSP), electrode reduction, electroencephalography (EEG), emotions

## Abstract

Most electroencephalography (EEG) based emotion recognition systems make use of videos and images as stimuli. Few used sounds, and even fewer studies were found involving self-induced emotions. Furthermore, most of the studies rely on single stimuli to evoke emotions. The question of “whether different stimuli for same emotion elicitation generate any subject-independent correlations” remains unanswered. This paper introduces a dual modality based emotion elicitation paradigm to investigate if emotions can be classified induced with different stimuli. A method has been proposed based on common spatial pattern (CSP) and linear discriminant analysis (LDA) to analyze human brain signals for fear emotions evoked with two different stimuli. Self-induced emotional imagery is one of the considered stimuli, while audio/video clips are used as the other stimuli. The method extracts features from the CSP algorithm and LDA performs classification. To investigate associated EEG correlations, a spectral analysis was performed. To further improve the performance, CSP was compared with other regularized techniques. Critical EEG channels are identified based on spatial filter weights. To the best of our knowledge, our work provides the first contribution for the assessment of EEG correlations in the case of self versus video induced emotions captured with a commercial grade EEG device.

## 1. Introduction

Emotions play an important role in many aspects of our daily lives, including understanding, learning, thinking, decision making, performing actions, and so on. Recent interest in the identification of neural correlations of emotion is motivated by diverse interests [1]. Emotion classification offers great assistance towards aiding people, for example, caring for disabled persons [2,3] and designing brain computer interfaces [4]. Electroencephalography (EEG) based emotion recognition might serve as a form of communication for the disabled [5]. Alternatively, it could be used to develop a human–computer interface to control neural stimulation [6]. Each of these potential applications poses unique requirements for research and requires a fairly high rate of emotion identification to be successful. 

Recalling memory of any specific incident, situation, or person can evoke excitement, anger, sadness, or grief. Though emotion evoked as a result of recalling any past memory may not be felt as strongly as the real experience, memories still play an important role in developing the psychology of a person and his behavior [7,8,9]. Along with positive emotions like satisfaction, pride, happiness, and so on, past memories can also activate negative emotions such as anger, frustration, envy, disgust, or fear [10,11,12]. It is quite significant to use a reliable and efficient emotion elicitation paradigm for emotion capturing experiments. There are different kinds of stimuli that are used in emotion research, for example, viewing images, listening to different genres of music, and watching videos. However, most of the emotion-based studies have used a single method to elicit emotions [12,13,14,15]. Thus, the question of “whether different stimuli of elicitation for same emotion generate any common subject-independent neural correlations” remains unanswered.

From the studies conducted in the domain of electroencephalography (EEG) based brain computer interfaces (BCI), it has been found that human emotions can be identified and discriminated from each other using EEG brain signals [12,13,14,15,16,17]. Although at the same time, we observe that studies on neural correlations of memory-evoked emotions, specifically fear, are scant. The EEG signals under different frequency bands have gained much research interest. Frequencies with lower ranges like alpha and mu are related to vigilance and movement, whereas high frequencies in EEG signals, such as gamma bands, are relevant to cognitive processes that include different emotions and feelings. In recent years, studies have continued to suggest connections between gamma band activities (GBA) and emotions [14]. One of the major goals while designing emotion recognition systems is to find the frequency bands that are most relevant to a specific emotional category. Li and Lu in one of their studies concluded that the gamma frequency band plays a major role in emotion recognition [18]. Dan Nie et al. also found that that higher frequency bands provide a more significant contribution to emotional responses when compared with lower frequency ranges [19].

Different combinations of feature extraction and classifiers are found in literature for the BCI systems targeting the classification of different brain states. Yu Zhang et al. [20] worked on an EEG based system for motor imagery, introducing a sparse Bayesian Extreme Learning Machine (SBELM) based technique for improvement in classification performance. Zhang Y et al. [21] proposed an algorithm, named temporally constrained sparse group spatial-pattern (TSCSP), as an extension of CSP to improve classification performance. The proposed technique is applied on three public EEG datasets. Jin et al. [22] worked on a sparse Bayesian method exploiting Laplace priors in a hierarchical manner under Bayesian evidence architecture. The proposed method is applied on two EEG datasets to boost classification performance.

In order to design a portable and compact BCI system, it is quite essential to keep a minimal number of EEG sensors or electrodes. To achieve this objective, several advanced algorithms were proposed, and we found a reduced number of electrodes in the BCI systems under consideration [23,24,25,26,27,28]. Some of the methods are iterative multi-objective optimization [29] and sequential floating forward selection (SFFS) [20], among others.

The presented work addressing the aforementioned issues focuses on human emotion recognition with two different ways of elicitation. Compared with well documented neurophysiological findings on emotions induced with effective stimuli, for example, videos and images, much less is known about self-induced emotions. The main contributions of this work are as follows:

- A new paradigm to induce emotion, specifically feelings of fear, with two different ways of elicitation: self versus audio/video clips has been proposed
- We attempted to identify if low-amplitude EEG signals acquired from neuro-paradigm based on emotional imagery or recalling past memories could be classified effectively with comparatively strong stimuli based on audio/video clips
- As CSP has been widely used in different scenarios of EEG based BCI applications, such as motor imagery, this work attempts to investigate whether it is also a good choice for emotion recognition.
- We compared conventional CSP with its regularized algorithms to investigate if classification performance could be improved
- By analyzing the spatial filter weight distributions, different electrode set configurations were found and, ultimately, subject independent electrode placement was obtained with a minor compromise on classification performance
- We confirmed that self-induced versus audio/video induced fear feelings exhibit subject-independent neural signatures at critical frequency bands and brain regions.

In this work, we worked on a new paradigm to induce emotion, specifically feelings of fear, with two different ways of elicitation: self versus video induced. In the case of self-induced or emotional imagery, subjects were asked to imagine or recall any memory to evoke emotions. In current fast moving life, people have come across unfortunate incidents, terrorist attacks, or sometimes any unfortunate event that has surely affected the normal mental and psychological state and memories. While in the second scenario, audio/video clips were shown to the participants to evoke feelings of fear. From the recorded EEG data, we extracted bandpass features from multichannel EEG data, spatial filters were obtained based on the CSP algorithm, and then we trained an linear discriminant analysis (LDA) classifier using these features as inputs. To perform spectral analysis, different setups for frequency bands were chosen and classification performance was compared in each of the selected bands. Additionally, six regularized CSP algorithms [30] were compared with conventional CSP, in order to address the issue of overfitting and noise observed while applying the CSP algorithm.

The rest of the paper is organized in the following manner. The research methodology is explained in Section 2. First, the whole experimental setup is explained with all the descriptions related to participants, experimental protocol, and EEG recording equipment. In the next stage, the methodology for data analysis of the collected EEG data is explained. Section 3 comprises the results and discussion, followed by conclusions in Section 4.

## 2. Materials and Methods

The proposed methodology to classify self versus audio/video clips induced emotions consists of four steps, that is, EEG data acquisition, preprocessing, feature extraction, and classification. First, electrical activity is recorded during the experiments from the human scalp using an EEG headset. The recorded EEG data are then preprocessed and band power features are extracted in different frequency bands in which the neurophysiological signals reside. The extracted features first train the classifier, and finally features from the test data are used by the classifier. The details of each block are described in the following subsections.

### 2.1. Experimental Setup

In the following subsections, details of the participants, the headset used for EEG data collection, and the experimental procedure are explained.

#### 2.1.1. Participants

The study was conducted in accordance with the Declaration of Helsinki and approved by the local Ethics Committee of Bahria University, Pakistan (ERC/ES/001). Fifteen young, university-going, healthy participants (seven female; eight male) with no psychological problem participated in the study. The mean age of the subjects was 22.57 years. Each participant was informed about the purpose of the study and signed informed consent prior to participation. Alarcao et al. [31] presented a detailed analysis and survey on EEG based emotion recognition studies that suggests the median for the number of subjects considered is 15 [31]. The survey covers almost one hundred papers for EEG based emotion recognition studies from the years 2009 until 2016. Keeping this observation in consideration, we took the subject pool to be comprised of 15 participants.

#### 2.1.2. EEG Recordings

**Figure 1:**
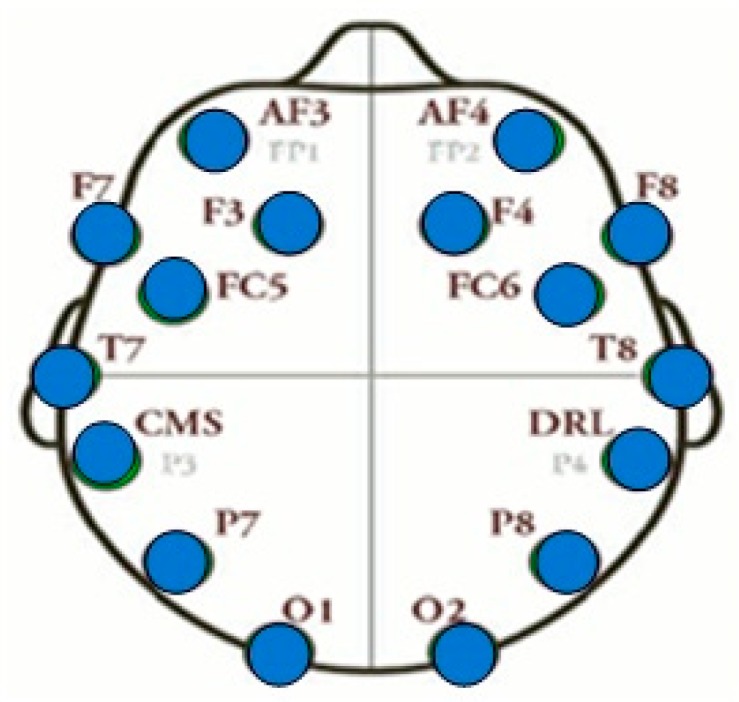
Electrode placement for Emotiv EPOC headset.

Commercial grade Emotiv EPOC EEG Headset was used for capturing brain signals during the experiments. It is a wireless headset that requires less placement time and effort and offers improved mobility and flexibility when compared with other medical grade EEG headsets [32,33,34,35,36]. The EEG signals were recorded with a sampling frequency of 256 Hz from the Emotiv EPOC headset with fourteen EEG channels, namely, AF3, AF4, F3, F4, FC5, FC6, F7, F8, T7, T8, P7, P8, O1, and O2, as shown in Figure 1. The data were recorded with the Emotiv provided software named ‘Emotiv Xavier Test bench’. Fourteen (14) active electrodes on the headset were arranged according to the 10-20 international system.

The name, 10-20 international refers, to the actual physical distances between neighbouring EEG electrodes. They are either 10% or 20% of the total front–back length of the skull or 10% or 20% of the right–left of one side. Each letter of fourteen electrodes identifies the lobe locations, while the corresponding number identifies the hemisphere location. The letters F, P, T, O, C, and P stand for frontal, parietal, temporal, occipital, central, and parietal lobes, respectively. Odd numbers refer to the EEG channels on the right hemisphere, while even numbers refer to those located towards the left hemisphere.

#### 2.1.3. Procedure

First of all, a briefing was given to the participants regarding the purpose of the study and they were informed that their brain activity (EEG) would be recorded while considering two different stimuli of inducing fear. Each participant was requested to fill in a questionnaire. The questionnaire asks participants to write down any event of their life/imagination that could induce feelings of fear. Participants were free to choose any scenario, and could write as little or as much detail as they liked. Participants were requested to write the detail as the series of scenes that could be recalled accordingly. Once the questionnaire was filled in, only those participants who gave their consent to partake in the experiments were shortlisted. The participants were called on different days as per a prepared schedule. At any time, only one participant was present to perform the experiment and a maximum of four participants were called in a single day.

While filling in the questionnaire, discussions were conducted with participants. They were asked to write down the scenario/imagination that could induce fear emotions. For example, some participants wrote down scenarios related to a particular phobia, for example, fear of height, spiders, and so on. Using this paradigm, although the way of inducing fear was different, the category of emotion remained the same for the participants. It is possible that the level of arousal for fear among the participants may vary. However, in our case, we have labeled our data in two classes; that is, +1 emotional fear state as self-induced and −1 emotional fear state as audio/video induced. This was done with reference to other studies in which the arousal level for the same emotion may be different among participants, but all the data samples for one emotion are classified as one class [12,14,27].

The experiments were conducted inside the room with controlled darker illumination level and quiet environment. For better connectivity, the hairs of the participant should be wet. Thus, first a small quantity of water was sprayed onto the participant and then the EPOC headset was placed over the head. On average, the whole activity of setting all the settings and getting the electrodes properly connected took 15 min.

The subject sitting on the chair with the EPOC headset mounted on his head was first asked to close their eyes. The experiment starts with 5 s of baseline recording. The researcher then asks the participant to recall the frightening event/imagination they mentioned in the questionnaire. They were requested to immerse themselves in that particular memory or imagination and recall as per the sequence of scenes specified in the questionnaire. First, they performed a few practice sessions. Participants were also requested to keep their face and body steady during the experiments. The researcher verbally signals the participant to start the activity and starts EEG recording using the EMOTIV Xavier Test bench. After 60 s, the researcher gives the signal to stop the activity and stops EEG recording. Following the block of Session I for self-induced, the participants were given a break of 30 s and asked to clear their mind.

Following the session for self-induced emotion with recalling memory, the second session for inducing fear with audio-video clip was conducted. During each block of this session, two videos were shown. One video for evoking fear, and a pleasant video shown to relax the participant during the resting state. Table 1 provides the list of videos shown to the participants in the given order.

**Figure 2:**
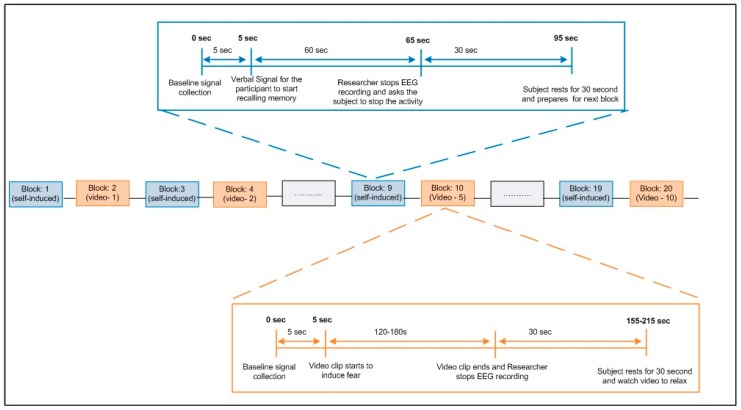
Block diagram of the experiment. EEG—electroencephalography.

The complete flow of the whole experiment is elaborated in Figure 2. Participants performed one block of Session-I (self-induced), then one block for Session-II (video induced) in an alternate manner. The procedure is repeated until ten blocks of both sessions are completed. The following is the sequence for each block.

**a.**  **Single block of Session-I (Self-induced)**
  1. 5 s of baseline signal collection
  2. Verbal signal was given to start the activity
  3. Participant recalled the same incident that they mentioned in questionnaire
  4. After 60 s, the activity stopped
  5. 30 s rest time
**b.**  **Single block of Session-II (Videos-induced)**
  1. 5 s of baseline signal collection
  2. Video clip starts and verbal signal was given to start the activity
  3. Display of the movie clip to induce fear (120–180 s)
  4. Video finished
  5. Display of the movie clip to relax (30 s)
  6. Video finished

Eventually, each participant carried out total twenty blocks. Out of these twenty, ten blocks were recorded for each of the two sessions—self-induced and videos-induced. The maximum time from the setting of the EEG headset and the experiments took one hour among all the participants. The whole protocol of the experiment is depicted in Figure 2. At the end of the experiment, we gathered feedback from each participant, asking if the participant found the whole experiment interesting and whether they were able to induce emotions while performing the experiments.

The duration from filling out the questionnaire until performing the experiments took a maximum of two weeks for all the participants. Because emotional imagery does not necessarily mean recalling a recent event/incident, the length of time may not pose any influence in emotional state or feeling level.

### 2.2. Data Analysis

**Figure 3:**
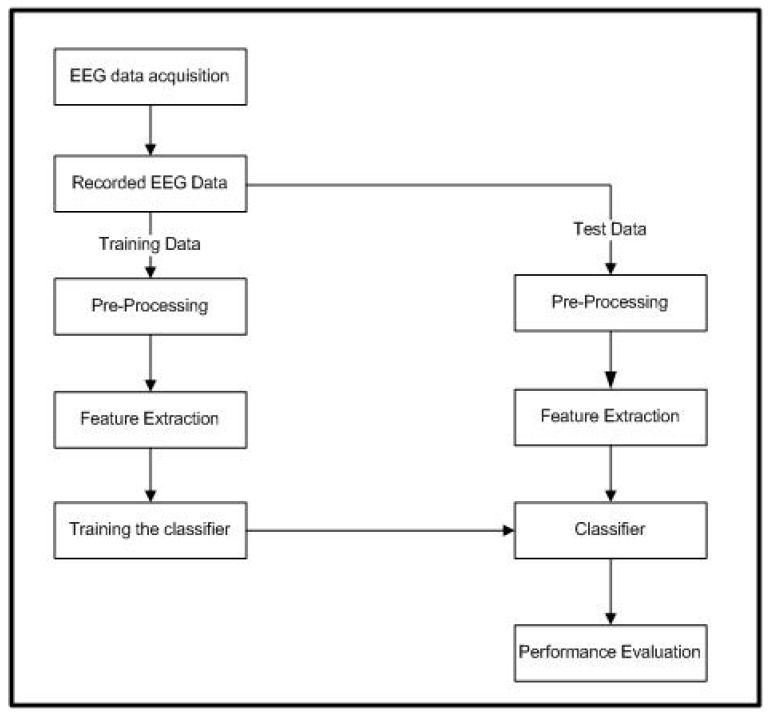
Methodology for data analysis.

The BCI system consists of two major phases. The first one is the learning phase, in which training data and their respective labels are used for training the classifier by mapping input data into its corresponding classes. The second phase uses this trained BCI system to predict the classes of testing/evaluation data. The predicted labels of the trained classifier are compared with the actual labels to evaluate the performance of the BCI system. In both phases, the methodology involves preprocessing, feature extraction, and classification. To perform the data analysis, this work used RCSP Toolbox designed in MATLAB [37]. The methodology we used to perform data analysis is shown in Figure 3.

#### 2.2.1. EEG Data Acquisition

As explained in Section 3.1, the experiments were performed firstly to acquire EEG data from all participants for the given scenario.

#### 2.2.2. Segmentation into Samples

This work considers discrete classification of the samples such that a class is assigned to each sample. For each block of both sessions, features are extracted from the time segment of the last 55 s, so that the data with intense emotional state will be considered for data analysis. For each block from Session-II, the last 55 s of the recorded EEG data from the video clip to evoke fear is considered. The EEG data are then segmented into non-overlapping time windows of one second (1 s) length. Data within each time window are referred to as a sample. Each sample x(t) was then associated with the label y(t) ∈ [+1, −1], corresponding to the self-induced fear as +1 and video-induced fear state as −1, respectively. This was done with reference to other studies in which the arousal level for the same emotion may be different among participants, but all the data samples for one emotion are classified as one class [12,14,27].

#### 2.2.3. EEG Preprocessing

The scalp recorded EEG data was bandpass filtered in seven different frequencies bands as follows: delta band (1–3 Hz), theta band (4–7 Hz), alpha band (8–13 Hz), beta band (14–30 Hz), low gamma band (31–50 Hz), high gamma band (50–100 Hz), and the whole range of 1–100 Hz. For each of these frequency bands, a Butterworth filter of order 5 was used. The Butterworth filter has been used previously in many EEG based emotion recognition studies [38,39,40,41]. This operation produced seven bandpass filtered datasets for each participant that served as input to the feature extraction stage.

The preprocessed data for videos only are now subjected to an artefact rejection procedure using independent component analysis (ICA). The independent components (ICs) with large weights over frontal or temporal areas, together with corresponding temporal course showing eye movement or muscle movement activities, were removed. Because Session-I had no eye blink artefacts, a higher number of ICs were rejected in the case of Session-II.

#### 2.2.4. Feature Extraction Based on CSP

Within the field of pattern recognition system, feature extraction plays an important role in developing a suitable presentation of data for the next stage of classification. In our work, we used band power features that are provided as input to the common spatial pattern (CSP) algorithm, a technique that is widely used to obtain spatial filters for classification of EEG data [42,43]. CSP generates a filter matrix ω that maximizes the variance of the projected signal for one class, while minimizing for the other. In this work, the data for fear that is self-induced are identified as one class, while the EEG data for fear evoked with videos are labeled as other class. The variance of the band-pass filtered signals is directly related to the signal power in that corresponding frequency band. The spatial filters obtained from CSP were optimized separately for each frequency band, such that the resulting CSP signals have maximal variance difference between the two emotional states in consideration [37]. CSP uses the spatial filters ω that optimize the following objective function:

(1) $$J\left(\omega \right)=\frac{\omega^{T}X_{1}{}^{T}X_{1}\omega }{\omega^{T}X_{2}{}^{T}X_{2}\omega }=\frac{\omega^{T}C_{1}\omega }{\omega^{T}C_{2}\omega }$$

where T refers to transpose, X_1_ and X_2_ are the data matrix for two classes with label y(t) ∈ [+1, −1], and C_1_ and C_2_ are the covariance matrices of each class.

In order to address this optimization problem, one can observe that if the filter ω is rescaled, the function J (ω) remains unaltered. This means that the rescaling of ω is arbitrary, such that J (kω) = J (ω) with k as a real constant. Subject to the constraint ω^T^C_2_ω = 1, extremizing J (ω) is equivalent to extremizing ω^T^C_1_ω, because there is always a possibility to find rescaling of ω such that ω^T^C_2_ω = 1 [34]. Based on the Lagrange multiplier method, the optimization problem can be addressed by extremizing the following:

(2) $$L\left(\lambda ,\omega \right)=\omega^{T}C_{1}\omega -\lambda \left(\omega^{T}C_{2}\omega -1\right)$$

As the spatial filter ω is extremizing L, the derivative of L with respect to ω becomes zero.

$$\begin{array}{c} \frac{\partial L}{\partial \omega }=\omega^{T}C_{1}\omega -\lambda \left(\omega^{T}C_{2}\omega -1\right)\\ \Leftrightarrow C_{1}\omega ={\lambda C}_{2}\omega \\ \Leftrightarrow C_{2}{}^{-1}C_{1}\omega^{T}=\lambda \omega \end{array}$$

Here, the standard eigen value problem is obtained, and the filters in Equation (1) are termed as the eigen vectors of C_2_^−1^C_1_. The spatial filters correspond to the largest and lowest values of these eigen vectors. In the case of the CSP algorithm, the logarithm of EEG signal variance after projection onto the spatial filters constitutes the features extracted [37].

For our data, the features are calculated on a basis of 1 s samples from the last 55 s of each block, resulting in 55 × 10 = 550 samples from the ten blocks each of the self- and video-induced sessions. Each extracted sample was then associated with a label y(t) ∈ [+1, −1], as explained before. Because the sampling frequency is 256 Hz, each sample is a matrix of 256 rows and 14 columns (as many number of electrodes). The segmentation into 1 s window length samples was implemented to generate enough samples for more efficient classification performance [14,44,45,46,47]. Wang et al. performed emotion classification from EEG signals using different time window lengths and concluded that 1 s time windows produce better results when compared with others [47]. To perform classification, the samples are divided into the training set and testing set. CSP operation is then applied on the training data and calculated bandpass features for both training and testing datasets, such that the former was fed to train the classifier and the latter was used to evaluate classification accuracy, as described in the next step.

Here, we extract features from the EEG dataset using the common spatial patterns (CSP) algorithm. EEG Signals is the multi-dimensional matrix for storing EEG data and their associated labels. The relevant information is given as follows with Varibales Considered:

Variables Considered.**Input:**
  EEG Signals: the EEG Signals from which extracting the CSP features. These signals are of a structure such that:
   EEG Signals.x: the EEG signals as a [Ns × Nc × Nt] Matrix where
      Ns: number of EEG samples per sample
      Nc: number of channels (EEG electrodes)
      Nt: number of samples
     EEG Signals.y: a [1 × Nt] vector containing the class labels for each sample
     EEG Signals.s: the sampling frequency (in Hz)
  CSP Matrix: the CSP projection matrix
    nbFilterPairs: number of pairs of CSP filters to be used. The number of features extracted would be twice the value of this parameter. The filters selected are the one corresponding to the lowest and highest eigenvalues.
**Onput:**
  Features: the features extracted from the above mentioned EEG data set as a
[Nt × (nb Filter Pairs × 2 + 1)] matrix, with the class labels as the last column.

### 2.3. Classification

The next major step to identify EEG neurophysiological signals in a BCI system is translating the features into interpretable commands by means of classification. In this phase, the set of features extracted from the EEG testing dataset are given as input to the classifier that assigns a class or label to it. In our work, linear discriminant analysis (LDA) was used as classifier, which is commonly used for solving binary as well as multiclass classification problems [48]. After extracting band power features using CSP, we fed the logarithm of variance of the samples as the band pass features into an LDA classifier. The classification performance of a BCI system can be estimated by calculating its accuracy, which is defined as the correspondence between classification output as predicted label and actual label (i.e., in our case, if the predicted emotion state matches the actual state). For each subject, the average classification accuracy was computed using a scheme of 10-fold cross-validation in which the samples are distributed into ten subsets. Out of ten subsets, nine are included in training dataset, while the remaining one is considered for testing purposes. The process is repeated for ten times with all the possible dataset splits. The classification accuracy is computed as the ratio of the correctly classified number of samples and the total number of samples.

For our data, the experiment comprised two sessions for evoking fear feelings. Session I is based on emotional imagery, while in Session II, audio/video clips are shown to the participants to evoke the fear emotion. For each session, ten blocks of 60 s are performed. Thus, in total, twenty blocks are conducted for each participant. As explained before, the features are calculated on a basis of 1 s samples from the last 55 s of each block, resulting in 55 × 10 = 550 samples from the ten blocks of Session-I and, in the same manner, 550 samples from the ten blocks of Session-II. Thus, we have a total of 1100 samples for each participant. Now, to perform ten-fold cross validation, the dataset is split in ten equal parts and 9 out of 10 folds are used as training data, while the remaining one is retained as the validation set so that in the end, every sample or instance has been used exactly once for testing purposes. For the testing set, one block from each session (55 + 55 = 110 samples) is considered, while the remaining blocks (990 samples) are used for training purposes. In the end, every block has been used exactly once for testing purposes.

### 2.4. Conventional CSP vs. Regularized Algorithms

In order to address the issues of sensitivity of CSP towards noise and overfitting, there is a need to regularize it. One way to regularize CSP is based on covariance matrix estimates, as these estimates are sometimes affected by noise and small training sets. Another way to address regularization is based at the level of objective function defined in Equation (1). In this method, regularization is performed by imposing priors on the filters to obtain [37]. It can be performed as follows:

$$\begin{array}{c} C_{a}=(1-\gamma )C_{b}+\gamma I\\ \mathrm{with}\;C_{b}=(1-\beta )S_{c}C_{c}+\beta G_{c} \end{array}$$

where $C_{c}$ is the initial spatial covariance matrix for class c, $C_{a}$ is the regularized estimate, I is the identity matrix, $S_{c}$ is a constant scaling parameter (a scalar), γ and β are two user-defined regularization parameters (γ, β € [0, 1]), and G_c_ is a generic covariance matrix.

Regularizing the CSP objective function, as mentioned in Equation (1), provides another method to achieve CSP regularization. In this approach, a regularization term is added to the objective function to penalize the solutions that do not satisfy the prior. Keeping this in consideration, the equation for objective function becomes the following:

(3) $$J_{P1}\left(\omega \right)=\frac{\omega^{T}C_{1}\omega }{\omega^{T}C_{2}\omega +\alpha P\left(\omega \right)}$$

where P (ω) is a penalty function. Its purpose is to measure how much a prior is satisfied with ω spatial filter. The more the spatial filter satisfies it, the lower the penalty function. Therefore, in order to extremize J_P1_(ω), the penalty function P (ω) needs to be minimized. For this purpose, the regularization parameter α is introduced, where α ≥ 0. A higher value of α will ensure the more satisfied prior [37].

Various regularized CSP algorithms are introduced [43] to cater for issues of overfitting and noise associated with CSP. One of the objectives of the work is to investigate which regularized algorithm of CSP achieves maximum classification accuracy. For this purpose, the following regularized CSP algorithms are compared:

- Conventional CSP (CSP) (already explained earlier)
- Composite CSP (CCSP)
- Composite CSP with Kullback–Leibler divergence (CCSP-KL)
- CSP with Tikhonov regularization (TR-CSP)
- CSP with weighted Tikhonov regularization (WTR-CSP)
- CSP with diagonal loading using cross validation (DL-CSP-auto)
- CSP with diagonal loading using cross validation (DL-CSP)

Composite CSP (CCSP1): Composite CSP algorithm introduced by Kang et al. [30] based on regularization of the covariance matrices from other participants’ data. The algorithm keeps in consideration a generic matrix that identifies a specific prior on how the covariance matrix should be for a given mental activity considered. In the case of composite CSP (CCSP1), this generic matrix is constructed as the weighted sum of the covariance matrices of other participants’ data corresponding to the same brain activity. Complete details of this technique are available in the work of [30].

Composite CSP with Kullback–Leibler divergence (CCSP2): This method was also proposed by Kang et al. [30]. The same criterion is used for construction of the generic matrix mentioned above, but here the weights are defined as per Kullback–Leibler (KL) divergence among the subjects.

CSP with Tikhonov regularization (TR_CSP): CSP with Tikhonov regularization (TR) or TR_CSP presents a classical form of regularization, initially applied in the case of regression problems that consist of penalizing solutions with large weights. This regularization technique is expected to constrain the solution to spatial filters with a small norm, thus mitigating the effect of artifacts and outliers [34]. Further explanation of this technique is found in the work of [37].

CSP with weighted Tikhonov regularization (WTR_CSP): CSP with weighted Tikhonov regularization presented by Fabien et al. [37] is based on the CSP objective function regularization using quadratic penalties. Using this approach, for each electrode, high weights are penalized equally. Because of different contributions of channels in spatial filters, penalty levels are defined based on information available in the literature for which region of the brain, and ultimately sensors placement, is more relevant to the specific mental activity [37].

CSP with diagonal loading (DL_CSPauto): Another form of covariance matrix regularization used in the BCI literature is diagonal loading (DL), which consists of shrinking the covariance matrix towards the identity matrix. Interestingly enough, in this case, the value of the regularization parameter can be automatically identified using Ledoit and Wolf’s method [49].

CSP with diagonal loading using cross validation (DL_CSP): This is similar to the previous one, DL_CSPauto. The only variation is that authors have used cross validation of regularization parameter to check the efficiency of this approach [49].

For each participant data, the above-mentioned (R) CSP filters were learnt on the training set available. Log-variances of the filtered EEG signals were then used as input features to an LDA classifier. The features based on different frequency bands are input to these algorithms and classification performance for each spectral band is evaluated.

### 2.5. Electrode Reduction

Keeping a minimal number of channels is essential for designing a portable brain–computer interface system for daily usage. To develop a daily use system, several advanced algorithms were proposed to reduce the number of electrodes in BCI by selecting some key EEG channels [23,24,25,26], for example, iterative multi-objective optimization [29] and sequential floating forward selection (SFFS) [23]. In this work, a method is applied to find the least number of EEG channels to achieve maximum classification accuracy. The approach is based on first identifying the spatial filter weights from CSP using a complete set of channels. In the next step, the channels are selected based on the maximal filter weights. Channels with highest values are selected and data only from these channels are used to find the accuracy in next run and, ultimately, the optimum combination of the number of channels with obtained classification accuracies is reported. The approach is based on our previously proposed methodology, as mentioned in the work of [50]. By performing this task, we try to identify if classification can be improved using fewer channels, as well as whether there are any common channels selected for different subjects by using this approach.

## 3. Results and Discussion

### 3.1. Classification Performance

The classification performance of a BCI system can be estimated by calculating its accuracy, which is defined as the correspondence between classification output as predicted label and actual label (i.e., in our case, if the predicted emotion state matches the actual state). For each subject, the average classification accuracy was computed using a scheme of 10-fold cross-validation in which the samples are distributed into ten subsets. Out of ten subsets, nine are included in the training dataset, while the remaining one is considered for testing purposes. The process is repeated ten times with different dataset splits. To avoid the bias of training and testing data, the whole block is considered. While performing 10-fold cross validation, one block from each of the two sessions is considered in the testing data. While the remaining 18 blocks are considered in the training set. The accuracies and results specified in the forthcoming tables and sections are based on the average of the obtained accuracies. The result of classification accuracies obtained is mentioned in Table 2. It is noteworthy that there are some participants whose results are greater than 75%, while some of the others results are less than 60%. This wide difference in accuracies reflects the difference of the underlying neurophysiological mechanisms among the subjects to feel and express emotions.

To perform the spectral analysis, input features are extracted from different bands, namely, delta band (1–3 Hz), theta band (4–7 Hz), alpha band (8–13 Hz), beta band(14–30 Hz), low gamma band (31–50 Hz), high gamma band (50–100 Hz), and the whole range of 1–100 Hz, as mentioned previously. The gamma band is further divided into lower and higher band ranges. Classification accuracies based on different frequency bands are mentioned in Table 2. The highest achieved accuracies for each subject are highlighted in the table. It is observed that the classification performance in gamma bands is obviously better than that of alpha, beta, delta, and theta bands. This observation partly reflects that high frequency bands play a more significant role in the emotion recognition system when compared with low frequency bands. Although for a few subjects, frequency bands other than gamma are also significant, such as for subjects 4, 5, 11, and 15, the beta band (14–30 Hz) provides better accuracy when compared with the gamma band.

Previous neuroscience studies, such as Li and Lu [18], have found that gamma bands of EEG are more relevant for emotion classification using pictures and images as stimuli. Dan Nie et al. also suggested that higher frequency bands contributed to human emotional response when compared with lower frequency bands [19]. Our findings are consistent with the existing results. Having the highest mean classification accuracy of 72.74% across subjects and single subject accuracies over 70% in 13 out of 15 subjects, these findings reasonably present evidence for feasibility of reliable classification of fear emotional state induced with two different stimuli.

While exploring EEG based emotion recognition studies, different feature extraction techniques have been used. Moreover, for classification purposes, there are algorithms other than LDA. In this study, we evaluate the effectiveness of CSP in combination with LDA for the classification purpose of the same emotions with different stimuli. CSP with LDA produced better results in other EEG based studies, but from the results presented here, it can be inferred that the combination of the two is effective in producing comparable results for emotion recognition as well.

As discussed before in Section 2.3, the CSP algorithm has overfitting and noise issues. In order to cater for these issues, regularized CSP algorithms are introduced. Here, we are going to find out which variant of CSP achieves maximum classification accuracy. For each participant data, the regularized (R) CSP filters were learnt on the training set available, as described in Section 2.3. Log-variances of the filtered EEG signals were then used as input features to the LDA classifier, as done in the case of conventional CSP. The features based on different frequency bands are input to these algorithms and the results are mentioned in Table 3.

#### Statistical Analysis

**Figure 4:**
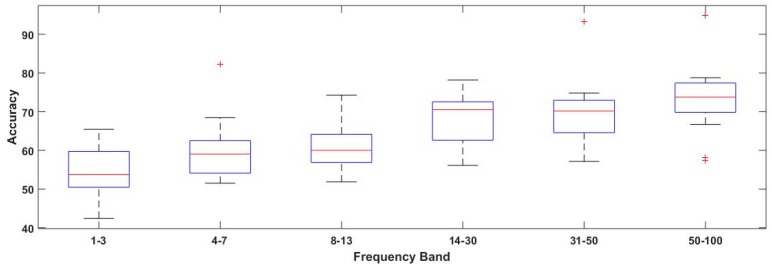
Box plot for classification accuracies with respect to frequency bands as obtained from Table 3. + symbol shows the outliers, while red line represents the median values.

**Figure 5:**
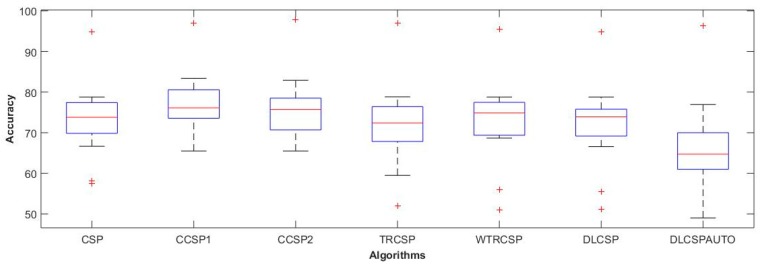
Box plot for classification accuracies with respect to different feature extraction techniques as obtained from Table 3. + symbol shows the outliers, while red line represents the median values. CCSP—composite CSP; TR_CSP—CSP with Tikhonov regularization; WTR_CSP—CSP with weighted Tikhonov regularization; DL_CSP—CSP with diagonal loading using cross validation.

The classification accuracies obtained in Table 3 with respect to frequency bands and algorithms were compared and visualized with the help of box plots, as mentioned in Figure 4 and Figure 5. From the visual inspection, it can be concluded that the gamma band is most suitable for the majority of the subjects, which is in consistent with the literature [18,51,52]. Moreover, the beta band also performed well next to the gamma bands. Figure 5 presents the comparison of mean accuracies achieved with conventional CSP in comparison with other regularized algorithms. Here, we observe that composite CSP (CCSP1) and composite CSP with Kullback–Leibler divergence (CCSP2) regularized techniques outperformed the conventional technique quite substantially, which supports the notion that when using CSP, regularization should be used in order to deal with its non-robust nature. While other variants including TR_CSP, WTR_CSP, DL_CSP, and DL_CSP_auto were not able to improve the accuracy, within the gamma band of 50–100 Hz, the mean accuracy obtained with conventional CSP was 72.74% (as per Table 2), while with CCSP1 and CCSP2, it increases to 76.97% and 75.95%, respectively.

In order to study the discrimination abilities of the recorded data, a one-way analysis of variance (ANOVA) test was performed on the extracted band-pass features. The difference between the mean of features in two different scenarios, self- versus video-induced, was found to be significant (*p* < 0.05). The significance of one-way ANOVA shows that there is at least a significant difference between the means of the samples from the two classes.

Furthermore, two-way analysis of variance (ANOVA) was performed for analyzing (1) the difference among the considered frequency bands and (2) the difference amongst feature extraction algorithms. The test results show significant difference with respect to the frequency bands p = 0.031 (*p* < 0.05), while no significant difference is observed between the algorithms p = 0.16

### 3.2. Electrode Reduction

**Figure 6:**
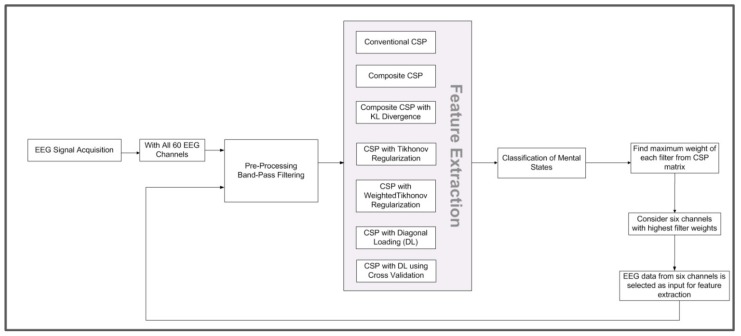
Methodology to find the reduced number of electroencephalography (EEG) channels based on CSP filter weights [50].

In order to design a portable and compact BCI system, it is essential to keep a minimal number of EEG sensors or electrodes. Minimizing the number of EEG electrodes not only helps in avoiding over-fitting and decreases computational costs, but it also enhances subject comfort and reduces headset setup time. In this work, we used spatial filter weights as the criterion to select EEG channels. This is with reference to our previous work, where we achieved better accuracies from only those selected channels based on CSP filter weights for motor imagery data [50]. For the selection of electrodes, a method is applied to find the lowest number of EEG channels to achieve maximum classification accuracy. The applied methodology is based on identifying the CSP spatial filter weights with a complete set of fourteen EEG channels as a first step. In the next stage, the channels with maximal filter weights are selected. Channels with the highest values are included to find the accuracy in the next run and, ultimately, the optimum combination of number of channels with obtained classification accuracies is reported, as mentioned in Figure 6. The approach is based on our previously proposed methodology, as mentioned in the work of [50]. By performing this task, we try to identify if classification can be improved using fewer channels, as well as whether there are any common channels selected for different subjects by using the above-mentioned approach. For motor imagery, we find ample studies conducted for optimal channel selection, as well as for emotion classification. However, specifically for emotions induced with different stimuli, the literature is scant. This work contributes in this respect as well.

#### 3.2.1. Subject-Dependent Channel Selection

**Figure 7:**
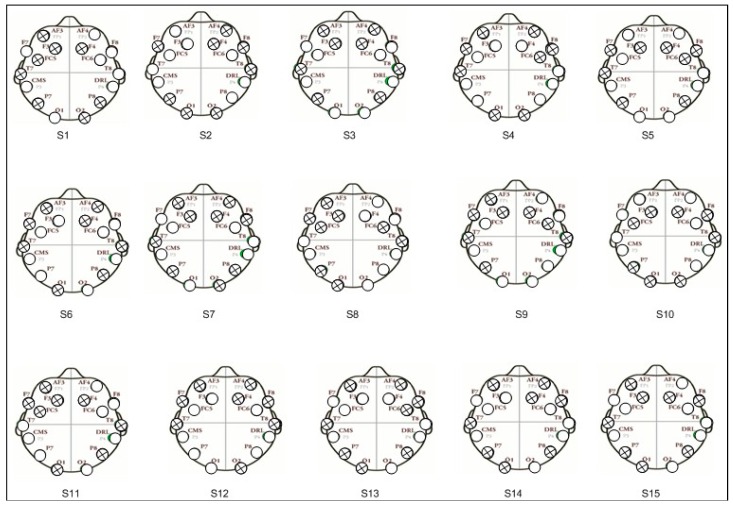
Specification of eight channels’ configurations for each subject.

On the basis of the spatial filter weights criterion, combinations of eight electrodes are obtained. Figure 7 displays configurations with eight channels for each subject. From the figure, it is obvious that the electrodes at frontal position AF3 and F4 are found in most of the subjects, indicating that there are some channels that have a higher frequency of appearance across subjects. This is in accordance with the already obtained results as mentioned in the works of [53,54,55]. It can be concluded that the frontal brain region is more relevant in the case of self-induced human emotions. The comparisons indicate that some brain regions are more useful for the discrimination between self- versus videos-induced fear state. Here, it is also interesting to observe that the channel configurations for almost all subjects are different even though the experimental protocol, feature extraction, and frequency band selection are same while performing the method for selection of channels. Our experimental results show that channel selection is predominantly subject-specific, as we observed in other research studies for EEG channel reduction [56,57].

**Figure 8:**
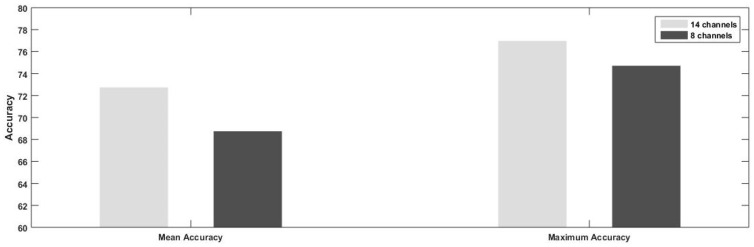
Comparison of mean and maximum classification accuracies achieved from subject-specific configuration of 8 and 14 electrodes, respectively.

In Figure 8, the results for maximum and mean accuracies achieved from a total of 14 and 8 channels are compared. It is observed that classification performance decreases with the reduced number of channels, in contrast to results that we obtained in our previous work, in which channel reduction using spatial filter weights is done for motor imagery data recorded with a total of 60 electrodes. In our previous work, we obtained better classification recognition by reducing the electrodes from 60 to 6 based on spatial filter coefficient values [50].

#### 3.2.2. Common Channels/Subject Independent Channels

In a BCI system, channel selection is closely related to classification performance. Thereby, channel selection is commonly conducted according to two criteria, that is, to yield the best classification accuracy by removing the noisy and irrelevant channels or to retain the least number of channels under the condition of keeping an acceptable classification accuracy. Because the classification accuracy is the most important criterion for evaluating BCI performance, the first criterion is mostly commonly employed in the research for channel selection.

In this study, we first collected signals of multichannel EEG for as many as 14 channels. Then, we found the critical channels and frequency bands through analyzing the spatial filter weight distributions. Li et al. pointed that the EEG data from irrelevant channels are irrelevant to emotion recognition tasks, and the weights of these channels tend to be distributed randomly [34,37]. Following this knowledge, we assume that the weights of critical channels tend to be updated to certain high values, which can represent how important they are for emotion recognition models.

On the basis of data obtained from Figure 6, we now identify the most frequent set of selected channels using the principle of weighted majority voting. These findings are significant for future development of EEG based emotion classification, because low-cost mobile EEG sensors with fewer electrodes are becoming popular for many new applications. Table 4 shows the number of channels that are selected subject-wise based on spatial filter weights. On the basis of the value of frequency of appearance of electrodes, we have selected configurations with a successive increasing number of electrodes from six to nine. For example, first we select six channels that have the highest frequency of appearance as AF3, F4, T7, P7, F3, and O1. Here, we also notice that there could be multiple possibilities as the electrodes F3, O1, and P8 have the same frequencies for appearance. On the basis of all these possible configurations, the mean accuracies over a varying number of channels across all subjects are shown in Table 5. The top two classification accuracies are highlighted in the table.

From Table 5, we find that the highest accuracy is achieved with configuration of these eight channels (AF3, F4, T7, P7, F3, O1, P8, and AF4), which equals 74.81%, while with all 14 channels, the highest accuracy achieved is 76.97%, as mentioned in Table 3. It shows that with the decreasing electrodes, we have to compromise on slight degradation in accuracy. The second highest accuracy is achieved with a configuration consisting of nine channels as mentioned in Table 5.

Now, we need to investigate the contrasting results that we obtained in our previous studies, where reduction in channels resulted in improvement in classification performance [50], while in current work, we experienced slight degradation. From the studies specifically for EEG channel selection [36,46], we observed that with data that already has a low number of channels, generally less than 15, sometimes we need to compromise on classification performance. Because Emotiv EPOC already has a small set of electrode configuration, with 14 channels that make it a commercial and portable device, if we are going to find a further reduced number of electrodes, we have to compromise on classification performance, as we have experienced in our case. It should be noted that although the 8-channel profile attains good enough classification performance in comparison to that of all 14 channels, the remaining 6 channels are not ‘uninformative’ for the emotion recognition task. Eight commonly selected channels able to produce a mean accuracy of 76.97% are mentioned. From this, it can be observed that out of eight electrodes, four reside in the frontal region. From the studies for channel reduction [27,46], we obtained similar results suggesting that the frontal region plays significant part in emotion recognition systems. Commonly selected channels with good classification performance suggest that neural signatures associated with self- versus video-induced fear emotion classification do exist and that they share commonality across individuals.

#### 3.2.3. Comparison to Related Work

It is hard to compare the obtained accuracy of individual emotional states with previous literature, because the number of targeted emotional states varies from study to study. Furthermore, comparisons become difficult as the related studies differ on several criteria. Few of these criteria are stimuli used for emotion elicitation, time period to record brain signals, the sensors used for EEG recording, and so on. To the best of our knowledge, there are only a few studies where emotional imagery is used for emotion classification. Moreover, no study is observed that has compared conventional CSP with its regularized techniques for improving classification performance in the case of self-induced emotions. Table 6 lists studies that are designed for emotion recognition. Moreover, few studies address the issue of channel reduction as well.

##### Computational Efficiency

While performing the spectral analysis, we achieved the best mean classification performance of 76.97% in the gamma band of 50–100 Hz. To determine the computational efficiency, we compare our results with similar studies and research works. Zhang et al. [27] have worked on the DEAP database containing EEG data for four human emotional states (relaxation, sadness, fear, and joy). The authors applied three different methodologies for the selection of EEG electrodes. A support vector machine (SVM) was used as the classifier. As per the obtained results, the authors achieved accuracy of 59.13% with a complete set of 32 electrodes and identified beta and gamma bands as being the most relevant for emotion recognition. Kothe et al. [12] in their work presented analysis of twelve subjects who were engaged in emotional imagery for love, frustration, anger, and so on. In this work, the authors were able to achieve an average accuracy of 71.3% using a filter bank common spatial pattern algorithm. In our proposed method, we achieved a mean classification of 76% with the gamma band. Similarly, Tamd et al. [14] and Li and Lu [18] found the same observation.

To the best of our knowledge, no study exists that targets improving the performance with CSP regularized algorithm in the case of EEG emotion recognition with multiple stimuli. However, looking at Kothe et al. [12], they have used a CSP invariant of filter bank CSP for EEG data from 250 electrodes of Bio Semi sensor and an achieved accuracy of 71.3%. Compared with this work, we obtained better performance with the composite CSP (CCSP1) regularized technique, increasing the performance from 72.74% to 76.97% in the high gamma band.

Furthermore, we see that Jatupaiboon et al. [49] conducted a study on emotion recognition induced using stimuli as Pictures of Geneva Affective Picture Database with Emotiv EPOC for EEG recording. SVM is used for binary classification of positive and negative emotions and achieved an accuracy of 85.41%. One of their findings for the most relevant frequency band is same as that of ours. Lacoviello et al. [17] worked on same paradigm of self-induced emotions, but with recalling unpleasant odor for the feeling of disgust. They used an eight-channel EEG sensor and found classification accuracy using only one channel. The best accuracy achieved with T8 channel is greater than 90%. One of the possible reasons for their highly improved classification recognition is the type of stimuli. They worked on single stimuli to induce emotions. Furthermore, the EEG recording device, feature extraction method, and classifier also differ from those used in our work.

##### Electrode Reduction

Discussing the issue of channel reduction, we find that Relief et al. [27] obtained 57.67% accuracy with 12 electrodes in comparison to 58.75% with 32 electrodes, which shows slight decrease in accuracy. Tamd et al. [14] achieved 83.99% accuracy with a complete set of 62 electrodes, while 12 channels provide better performance of 86.65%. Wang et al. [36] conducted a study for arithmetic task classification and achieved more or less the same accuracies with 14 and 4 channels, respectively. In this work, we obtained an accuracy of 74.81% with eight channels in comparison with 76.97% with all 14 channels, showing a slight decrease in accuracy. From the aforementioned studies, along with others, we observed that because Emotiv EPOC already has a small set of electrode configuration with 14 channels, if we are going to find a further reduced number of electrodes, in some cases, we have to compromise on classification performance. Although with studies having at least 25 channels, the reduction of electrodes mostly favors the improvement of classification.

Concerning the electrode locations playing a significant part in classification, we attempted to find reduced electrode configuration. The results indicate that the highest accuracy is achieved with configuration of these eight channels (AF3, F4, T7, P7, F3, O1, P8, and AF4), where four out of nine channels reside in the frontal region. Jatupaiboon et al. [52] also identified fontal electrodes, especially F7 and F8, significant for emotion recognition. Zhang, J., et al. [27] performed emotion classification of four different emotional states of joy, fear, sadness, and relaxation. On the basis of the obtained results, they concluded that electrodes at frontal and parietal locations provide more discriminative information for emotion recognition. Tamd [14] worked on classification of positive, neutral, and negative emotions induced with video clips shown to the participants. They identified electrodes at the lateral temporal and prefrontal locations as being more important for emotion classification.

From the above-mentioned comparison with the other EEG based studies, we can infer that our proposed method has acceptable classification performance. While comparing our results with other EEG based emotion recognition systems, we find different ranges for accuracies. Some are above 80%, while a large number of studies produce results between 70% and 75%. As we are working on a different new paradigm, an exact comparison with other studies is not possible, but having a mean classification accuracy above 75% shows that our method is able to produce good comparable results with other studies.

#### 3.2.4. Limitations

The limitations associated with the study are identified as follows. Only one emotional state of fear is considered in this work. Two conditions are identified self-induced fear and the other is audio/video-induced. On the basis of the level of arousal, further class labels can be identified. The scope of subjects is young, healthy, university-going students. For EEG recording, a commercial grade low resolution device is considered.

## 4. Conclusions

Research on EEG signals based emotion recognition has achieved notable progress in past years. Previous studies have mainly focused on emotions induced with external single stimuli like videos, images, while a few studies focused on the classification of self-induced emotions. The presented work addresses the research question of whether different stimuli for the same emotion elicitation generates any common or subject-independent EEG correlations. A novel multi-modal emotion elicitation paradigm is presented. EEG data of fifteen young, healthy, university-going human subjects have been considered to recognize human fear emotions. Henceforth, the results manifest the correlations for this age group. The presented research work lays a significant foundation for emotion recognition with different scenarios of stimuli under consideration. To cater for the limitations, this work can be extended further in future. Emotional states other than fear, like anger, disgust, and joy, can also be considered. More subjects with different age groups can help in further generalizing our results. Furthermore, the same experiments could be performed with a medical grade EEG device to find if the classification performance could be improved further.

## Figures and Tables

**Table 1:** List of videos shown to the participants.

S. No.	Videos	Emotional State	Video Length (s)
1.	Lights out movie trailer	Fear	180
2.	Best Vacations: Jumping	Pleasant	30
3.	Video clip from Insidious movie	Fear	120
4.	Caught red-handed	Pleasant	30
5.	Conjuring official trailer	Fear	125
6.	Stunning China-UNESCO World Heritage	Pleasant	30
7.	Die in Disaster Movies	Fear	145
8.	Tourism Sites In Pakistan	Pleasant	30
9.	Scene from The Eye-Horror movie	Fear	120
10.	Berlin City Tour	Pleasant	30
11.	Snakes catcher in Indian forest	Fear	80
12.	BBC nature documentary 2016	Pleasant	30
13.	Female Restroom-Horror clip	Fear	180
14.	Nat Geo Wild HD Ocean of Giants	Pleasant	30
15.	Frightening Creepy Clown	Fear	130
16.	10-month-old babies	Pleasant	30
17.	Scene from The Conjuring 2	Fear	120
18.	Roller Coaster & Candy Coaster	Pleasant	30
19.	Fear of Snakes	Fear	125
20.	Army Man surprises his 8-year-old daughter	Pleasant	30

**Table 2:** Classification accuracies for each subject using common spatial patterns (CSP) for all considered frequency bands.

Subjects	Delta (1–3 Hz)	Theta (4–7 Hz)	Alpha (8–13 Hz)	Beta (14–30 Hz)	Low Gamma (31–50 Hz)	High Gamma (50–100 Hz)	(1–100 Hz)
S1	50.70	66.35	62.09	72.56	70.16	**73.90**	65.88
S2	53.70	59.26	55.56	70.37	**70.52**	66.67	66.67
S3	64.81	57.41	51.85	68.52	64.41	**70.41**	64.41
S4	62.96	59.26	59.32	**72.50**	65.00	69.64	69.64
S5	58.93	53.70	64.29	**71.64**	66.07	70.81	64.81
S6	53.70	55.36	61.11	61.11	**71.81**	70.91	70.91
S7	65.45	53.70	56.36	61.82	63.64	**74.07**	**74.07**
S8	57.50	62.96	55.56	61.11	73.81	**75.00**	71.00
S9	47.50	53.00	58.40	78.19	74.81	**78.21**	59.40
S10	53.56	61.22	65.29	56.12	57.14	58.18	**67.87**
S11	45.00	51.50	60.00	**65.00**	63.00	57.50	61.00
S12	50.39	59.04	63.82	71.80	70.35	**78.77**	64.42
S13	42.39	58.07	58.53	70.54	66.84	**78.34**	67.34
S14	60.00	82.22	71.11	75.56	93.33	**94.81**	87.41
S15	56.31	68.46	74.27	**77.18**	73.31	73.80	70.89
**Mean value**	54.86	60.10	61.17	68.93	69.61	**72.74**	68.38

**Table 3:** Mean classification accuracies using conventional and regularized CSP algorithms for all the considered frequency bands. CCSP—composite CSP; TR_CSP—CSP with Tikhonov regularization; WTR_CSP—CSP with weighted Tikhonov regularization; DL_CSP—CSP with diagonal loading using cross validation.

Algorithm/Frequency Band	CSP	CCSP1	CCSP2	TR_CSP	WTR_CSP	DL_CSP	DL_CSP_auto
(1–3 Hz)	54.86	65.00	63.93	52.54	53.51	49.49	51.62
(4–7 Hz)	60.10	65.85	66.30	61.30	62.05	60.91	60.52
(8–13 Hz)	61.17	64.22	63.77	60.32	59.27	59.46	59.79
(14–30 Hz)	68.93	**71.38**	**71.21**	69.24	69.05	66.76	68.08
(31–50 Hz)	69.61	72.49	72.19	69.48	70.19	69.91	68.77
(50–100 Hz)	72.74	**76.97**	**75.95**	72.06	72.86	72.20	66.28

**Table 4:** Frequency of channels appearance for each subject based on spatial filter weights.

Subjects	AF3	F7	F3	FC5	T7	P7	O1	O2	P8	T8	FC6	F4	F8	AF4
S1	√		√	√	√	√		√	√			√		
S2		√				√	√	√			√	√	√	√
S3	√		√		√	√			√	√		√		√
S4	√	√			√		√	√			√		√	√
S5		√	√		√	√			√		√	√	√	
S6	√	√			√		√		√	√		√		√
S7	√		√		√	√		√	√			√	√	√
S8	√	√	√	√		√	√			√	√			
S9	√	√	√		√	√				√	√	√		
S10			√	√		√	√	√		√		√	√	
S11	√	√	√	√			√		√			√		
S12	√	√	√		√			√		√		√	√	√
S13	√				√	√	√		√		√		√	√
S14	√				√	√	√		√			√	√	√
S15	√		√		√	√	√		√			√		
Frequency for appearance	12	8	9	4	11	11	9	6	9	6	6	12	8	8

**Table 5:** Mean accuracies over varying number of electroencephalography (EEG) channels.

No. of Electrodes in Selected Configuration	Possible Configurations	Mean Accuracy Achieved
6	AF3 F4 T7 P7 F3 O1	64.49
	AF3 F4 T7 P7 F3 P8	70.35
	AF3 F4 T7 P7 O1 P8	66.86
7	AF3 F4 T7 P7 F3 O1 P8	65.01
8	AF3 F4 T7 P7 F3 O1 P8 F8	69.94
	AF3 F4 T7 P7 F3 O1 P8 AF4	**74.81**
	AF3 F4 T7 P7 F3 O1 P8 F7	71.67
9	AF3 F4 T7 P7 F3 O1 P8 F8 F7	**74.45**
	AF3 F4 T7 P7 F3 O1 P8 F8 AF4	67.92
	AF3 F4 T7 P7 F3 O1 P8 F7 AF4	71.15

**Table 6:** List of studies using EEG signals to perform emotion recognition and other tasks. SVM—support vector machine; LDA—linear discriminant analysis.

Studies/Year	Type of Study (Emotion Recognition/Others)	Classifier	EEG Device with Total Number of Electrodes	Classification Performance	Relevant Frequency Band/Brain Regions
Zhuang et al. [58]	Self-induced emotion recognition (joy, neutrality, sadness, disgust, anger, and fear)	SVM	g.HIamp System with 62 electrodes	54.52%	High frequency rhythm from electrodes distributed in bilateral temporal, prefrontal, and occipital lobes produced outstanding performance.
Jatupaiboon et al. [52]	Emotion recognition two emotions (i.e., positive and negative)	SVM	Emotiv (14 electrodes (7 pairs))	With all channels: 85.41% With five pairs or ten electrodes 84.18%	Gamma band
Zhang et al. [27]	four emotional states (joy, fear, sadness, and relaxation)	SVM	32	Originally 32 channels Reduced to 8 channels with 58.51% versus the best classification accuracy 59.13%	It can be found that the high frequency bands (beta, gamma) play a more important role in emotion processing.
Zheng et al. [14]	positive, neutral, and negative	kNN, logistic regression, SVM, and deep belief networks (DBNs)	ESI Neuroscan with 62 channels	With all 62 electrodes, DE features and SVM classifier obtained accuracy of 83.99% 4 channels: degradation 82.88% 6 channels 85.03%	Beta and gamma bands
Kothe et al. [12]	Self-induced emotion: positive vs. negative	Logistic Regression	Bio Semi 250 gel based 250 electrodes	71.3%	-
Chanel et al. [16]	Memory recall: negatively excited, positively excited, calm-neutral states	LDA, Linear SVM, Prob. Linear SVM, RVM	Bio Semi Active II System with 64 electrodes	63%	-
Lacoviello et al. [17]	self-induced emotions: disgust vs. relax	SVM	EnobioNE 8 channels	With T8 channel only accuracy above 90%	-
Li and Lu [18]	Happiness vs. sadness	Linear SVM	62 channel	93.5%	Gamma Band (30–100 Hz)
Wang et al. [36]	Arithmetic mental task	SVM	14 Emotiv EPOC	97.14% with 14 electrodes. 97.11% with four electrodes	-
Author’s work	Fear emotion recognition: self- vs. video-induced	LDA	14	76.97 with all 14 channels 74.81 with 8 channels	High gamma and beta band
